# Metformin and rapamycin are master-keys for understanding the relationship between cell senescent, aging and cancer

**DOI:** 10.18632/aging.100561

**Published:** 2013-05-25

**Authors:** Vladimir N. Anisimov

**Affiliations:** Department of Carcinogenesis and Oncogerontology, N.N.Petrov Research Institute of Oncology, St.Petersburg, 197758 Russia

During the last decade it was established that conservative growth hormone/ insulin-like growth factor-1 (IGF-1) and target of rapamycin (TOR) signaling pathways plays a key role in the control of aging and age-associated pathology in yeast, worms, insects and mammals [1-3]. mTORC1 (mammalian target of rapamycin complex 1) is activated by insulin and related growth factors through phosphatidylinositol-3-OH kinase and AKT kinase signaling and repressed by AMP-activated protein kinase, a key sensor of cellular energy status [1]. mTORC1 involved into promotion messenger RNA translation and protein synthesis through ribosomal protein S6 kinases (S6Ks) and 4E-BP protein. mTORC1 also stimulates lipid biosynthesis, inhibits autophagy, and through hypoxic response transcription factor HIF-1α regulates mitochondrial function and glucose metabolism. The lifespan of S6K1 deficient female mice increased by 19% without effect on tumor development [see 1]. These data suggest that S6K1 plays a relevant role in lifespan regulation downstream of TORC1. Lamming et al. [4] have shown that decreased mTORC1 signaling is sufficient for lifespan prolongation independently from changes in glucose homeostasis. Rapamycin suppresses mTORC1 and indirectly mTORC2 that leads to metabolic lesions like glucose intolerance and abnormal lipid profile [1]. Treatment with rapamycin or its more soluble form rapatar increased the mean lifespan in various strain of mice [1-3,5-7]. It is worthy to note that the regulation of growth hormone and IGF-1, oxidative stress, DNA damage, and metabolic pathways by calorie restriction could simultaneously leads to its anti-aging and anti-tumor activities as well as to reduction of the number of senescent cells in some tissues [1,3].

It was shown that the treated with antidiabetic biguanide metformin diabetes type 2 patients have from 25% to 40% less cancer than those who receive insulin as therapy or treated with sulfonylurea drugs that increase insulin secretion from the pancreas [1-3]. It was shown that biguanides like inhibitor of mTOR rapamycin prolong the lifespan of animals from yeast to mammals [2,3]. It means that the targets as well as signaling pathways and regulatory signals are also similar. Moreover, there is also the sufficient similarity in patterns of changes observed during normal aging and the process of carcinonogenesis. There is a mounting evidence for the similarity between the aging and carcinogenesis in response patterns of these two signaling pathways to pharmacological intervention.

**Figure 1:**
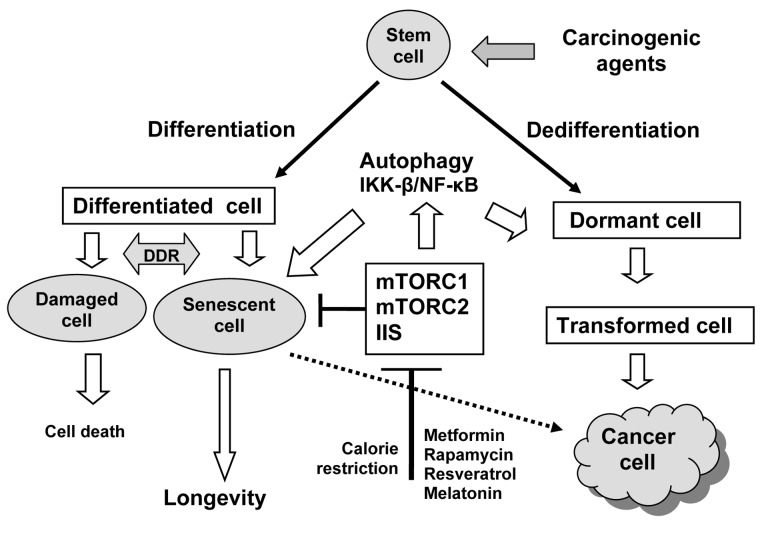
Relationship between aging and carcinogenesis: the key role of insulin/IGF-1-like signaling (IIS) and mTOR signaling DNA damage induced by environmental and endogenous factors (ROS, chemicals, ionizing radiation, ultra-violet, constant illumination, oncogenes, some diets, etc.) may leads to cellular senescence or cellular lesions which could be deleted by apoptosis or autophagy. The same agents can induce damages which followed by neoplastic transformation thus leading to cancer. Metformin, rapamycin, and some other compounds with mTOR- and IIS-inhibitory potential (resveratrol, melatonin) are able to modify both the aging and carcinogenesis.

DNA damage response signaling seems to be the key mechanism of establishment and maintenance of the senescence programme as well as of the carcinogenesis [1-3]. The available data on cellular senescence *in vitro* and on accumulation in cells in *vivo* of various premalignant lesionsprovide evidence suggesting that senescence is effective natural cancer-suppressing mechanism [1,2]. At the same time, adequate clinical application of therapy-induced ‘accelerated senescence’ for prevention or recurrence of human cancers is still not enough understood. The mechanisms underlying the bypass of senescence response in the progression of tumors should to be discovered. Recent studies reveal a negative side of cellular senescence, which is associated with the secreted inflammatory factors, and may alter the microenvironment in the favor of cancer progression designated as syndrome of cancerophilia or senescence-associated secretory phenotype (SASP) [1-3]. Thus, cellular senescence suppresses the initiation stage of carcinogenesis but is the promoter for intitated cells. We believe that the similarity of two fundamental processes - aging and carcinogenesis, - is a basis for understanding the two-side effects of biguanides and rapamycin on its (Figure 1). Recent finding provide the evidence of inhibitory effect of metformin and rapamycin on the SASP interfering with IKK-β/NF-κB [1] - an important step in hypothalamic programming of systemic aging [8]. It is remains to be shown whether antidiabetic biguanides and rapamycin can extend lifespan in humans.
